# A method to assess algicidal activity of microalgal extracts coupling microalgae produced in stirred closed photobioreactor operating in continuous with pulse amplitude modulated (PAM) fluorometry

**DOI:** 10.1016/j.mex.2020.101037

**Published:** 2020-08-19

**Authors:** Eva Menguy, Vincent Dumontet, Noémie Coulombier, Vincent Meriot, Loïc Le Déan, Vanille Barthelemy, Thierry Jauffrais

**Affiliations:** a Ifremer, IRD, Univ Nouvelle‐Calédonie, Univ La Réunion, CNRS, UMR 9220 ENTROPIE, 101 Promenade Roger Laroque, 98897 Noumea, New Caledonia; b Université Paris Saclay, CNRS, UPR 2301, ICSN, 1 Avenue de la Terrasse, F-91198 Gif-sur-Yvette, France; c ADECAL Technopole, 1 bis rue Berthelot, 98846 Noumea, New Caledonia

**Keywords:** Bioassay, Cytoxicity, Microalgae, Quantum efficiency, Fv/Fm, Non photochemical quenching, NPQ, electron transport rate, Phytochemistry, Anti algal

## Abstract

We describe in the present study a quick and reliable method based on chlorophyll a fluorescence to assess putative algicidal effect of different microalgal extracts. We couple microalgal production under chemostat cultivation mode to continuously produce a given microalgae species (e.g. *Dunaliella salina* in this study) at a stable physiological state to ease comparison between extracts tested; with a non-destructive method based on chlorophyll a fluorescence. Pulse amplitude modulated (PAM) fluorometry was used to assess over time the effect of different microalgal crude extracts on the efficiency of the photosystem II (PSII) of a tested microalgae (*Dunaliella salina*).

• Microalgal production at stationary phase in stirred closed photobioreactor (PBR) operating in continuous have stable photophysiological parameters, which is a prerequisite to compare the impact of different algicidal compounds.

• The combination of both methods, allows to quickly assess the algicidal effect of diverse microalgal (crude) extracts on the PSII efficiency of a tested microalgae.

• The method may be used to identify and isolate algicidal molecules affecting algal PSII using a bio-guided isolation protocol.

## Graphical abstract

**Figure:**
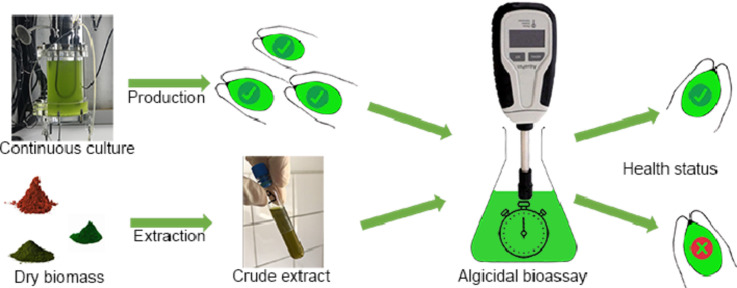

Specifications table

**Table:** 

Subject Area	Agricultural and Biological Sciences
More specific subject area	*Phycology, marine biology, biotechnology, algicidal activity, anti-algal activity*
Method name	*Assessment of algicidal effect using PAM fluorometry*
Name and reference of original method	*Long, M., Tallec, K., Soudant, P., Lambert, C., Le Grand, F., Sarthou, G., Jolley, D., Hégaret, H., 2018. A rapid quantitative fluorescence-based bioassay to study allelochemical interactions from Alexandrium minutum. Environ. Pollut. 242, 1598-1605.*https://doi.org/10.1016/j.envpol.2018.07.119
Resource availability	*-*

## Method details

Pulse amplitude modulated (PAM) fluorometry [1] is a non-destructive method based on chlorophyll *a* fluorescence that was already successfully used in previous study to assess allelochemical interaction between microalgae [2], [3], [4], algicidal / antialgal activity of marine bacterium [5] and herbicide activity of chemicals (e.g. Diuron [6]). However, photophysiological parameters acquired using PAM fluorometry such as the maximum quantum efficiency of the photosystem II (Fv/Fm) reflects photochemical processes that depend upon chloroplast reactions that use ATP and reductants provided by photosynthesis. It may therefore also be sensitive to cell energy metabolism and interactions between carbon and nitrogen assimilation. The use of microalgae produced in batch culture to carry out such experiment [2,^5^,6] may thus be controversial as these unbalanced culture conditions (e.g. pH, light, nutrient availability) continuously modify the microalgae photophysiology. It may induce bias such as an increase sensitivity to environmental stress or it requires to continuously produce batch cultures to have microalgae in similar physiological state. Nevertheless, Fv/Fm is frequently use to monitor environmental or nutritional stress of microalgae grown in batch cultures [7], [8], [9].

Here, we report the use of PAM fluorometry coupled with microalgal production of *Dunaliella salina* under chemostat cultivation mode to assess algicidal effect of different microalgal extract. The objective is to couple the continuous production of a given microalgae species (*D. salina*) under balanced condition with PAM fluorometry to (i) produce microalgae at a stable photophysiological state over many days [10], (ii) to avoid bias owing to unbalanced batch culture condition, (iii) to allow comparison between different algicidal extracts and finally (iv) to assess over time the impact of different microalgal crude extracts with a potential algicidal activity on the efficiency of the photosystem II of a tested microalgae (*D. salina*).

## Production of microalgal extracts

**Fig. 1:**
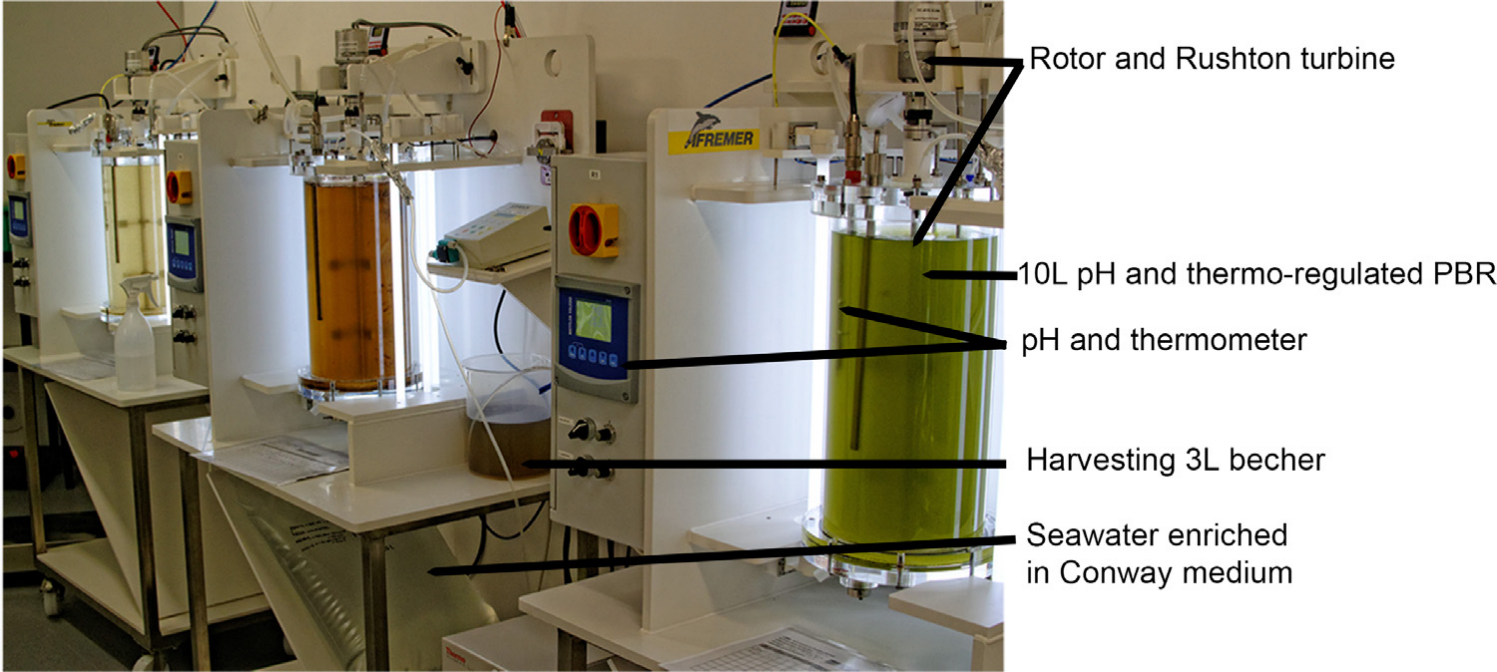
10 L stirred closed photobioreactors used to produce the eleven microalgae strains. Temperature, light, pH, rotor speed, dilution rate and medium content are controlled.

Eleven microalgal strains were used to test the method. The different strains (*Picochlorum* sp., *Odontella* sp., *Tetraselmis* sp.1, *Tetraselmis* sp.2, *Chaetoceros* sp., *Pavlova* sp., *Thalassiosira* sp., *Arthrospira* sp., *Nitzschia* sp., *Nephroselmis* sp. and *Entomoneis* sp.) were from different order and classes and all microalgal strains were produced in 10L photobioreactor (460 mm × 250 mm, Fig. 1) in transparent polymethylmethacrylate (PMMA) and operating in continuous [11], as described below.

At steady state, the microalgae were harvested daily until the desired biomass was collected (2 g of freeze dry microalgae). The medium was centrifuged (4 °C, 3552 g, 10 min), the supernatant discarded and the pellets lyophilized and stored at −80°C until extraction.

## Extraction in absolute ethanol

**Fig. 2:**
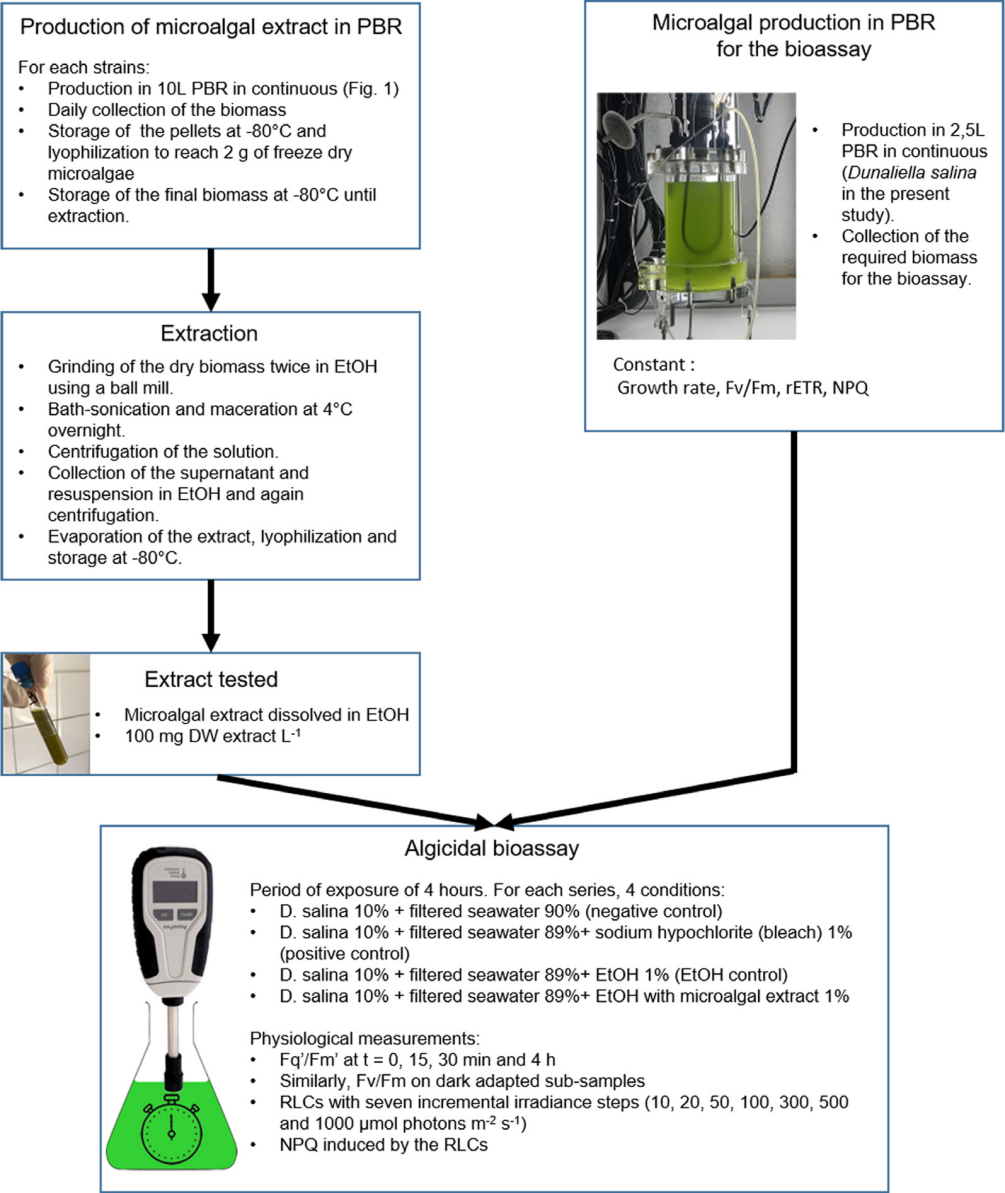
Schematic representation of the algicidal bioassay: from microalgae production to physiological measurements. PBR, photobioreactor; Fq′/Fm′ and Fv/Fm, Effective and maximum quantum efficiency of the PSII; RLCs, rapid light curves; NPQ non-photochemical quenching.

The microalgae extraction was carried out in absolute ethanol (EtOH),

1. ~2 g of freeze dry microalgal biomass (exactly weighed) were grounded twice (2 min, 30 Hz) in 10 mL of EtOH using a ball mill (Retsch, MM400).
2. The extract was transferred into a 50 mL Falcon tube and the mill was rinsed with 5 mL of EtOH. A microscopic observation was done to confirm cellular lysis. If necessary, to improve cellular lysis, extracts were bath-sonicated for 15 min and kept at 4 °C overnight.
3. The solution was centrifuged (4816 g, 15 min, 20 °C), the supernatant collected and the pellet re-suspended and homogenized in 10 mL of EtOH and again centrifuged (4816 g, 15 min, 20 °C, twice in total).
4. The extract was evaporated under reduced pressure using a rotary evaporator at 30 °C, transferred into a 15 mL pre-weighted Falcon tube and gently evaporated under nitrogen.
5. Finally, the extract obtained was lyophilized (−52 °C, 0.021 mbar) to evaporate the residual water, weighted and stored at −80 °C (Fig. 2).

## Bioassay

The targeted microalgae chosen for the algicide bioassay, *Dunaliella salina*, was selected owing to its use in similar studies [4,12].

1. The inoculum was cultured in batch in 200 mL filtered seawater (salinity 35‰, 0.2 µm) enriched in Conway medium [13].
2. The cultures were exposed to a continuous photosynthetic photon flux density (PPFD) of 90 µmol photons m^−2^ s^−1^. At the end of the growth phase 100 mL of the culture was used to inoculate the photobioreactor (PBR).

To ensure a daily production of microalgae in similar physiological condition, continuous culture was carried out in a 2.5 L PBR in PMMA. The pH and the temperature were regulated respectively at 8.2 using CO_2_ addition and 26°C via thermal exchange (stainless steel tube connected to a cold-water producing system). They were set on an Arduino electronic card and followed with a Raspberry computer. The PBR was continuously exposed to 90 µmol photons m^−2^ s^−1^ using neon light tubes on one side. A Rushton turbine (80 rpm min^−1^) was used to homogenize the culture medium (Fig. 2).

1. The PBR was sterilized with 5‰ peroxyacetic acid and rinsed twice with filtered seawater (0.2 µm) before being inoculated with 100 mL of *D. salina* in enriched filtered seawater [13].
2. The PBR was maintained for four days in batch culture and then alimented continuously to reach a daily renewal rate of 30%.
3. At steady state, the algae were used to assess the effect of the different microalgal extracts on their photophysiology.

*Dunaliella salina* was exposed for 4 h to microalgal extract dissolved in EtOH at 100 mg DW extract L^−1^, in 100 ml Erlenmeyer flasks (Fig. 2). For each serie, 4 conditions were tested:

• D. salina 10% + filtered seawater 90% (negative control)
• D. salina 10% + filtered seawater 89%+ sodium hypochlorite (bleach) 1% (positive control)
• D. salina 10% + filtered seawater 89%+ EtOH 1% (EtOH control)
• D. salina 10% + filtered seawater 89%+ EtOH with microalgal extract 1%

## Physiological measurements

The efficiency of the *D. salina* photosystem II (PSII) was followed and monitored using the effective and maximum quantum efficiency of the PSII (Fq′/Fm′ and Fv/Fm), rapid light curves (RLCs) and non-photochemical quenching (NPQ) to provide a proxy of the photophysiological state of *D. salina* after exposure to the microalgal extracts. Photosynthetic measurements were performed by pulse amplitude modulated (PAM) fluorometer (AquaPen-C AP 110-C, Photo Systems Instruments) with a blue light (455 nm).

1. Fq′/Fm′ was measured in triplicate at *t* = 0 min, 15 min, 30 min and 4 ± 1 h, with a saturating pulse (3000 µmol photons m^−2^ s^−1^), according to the equation [1,14]:
  (1) $$\frac{{\text{Fq}}^{\prime }}{{\text{Fm}}^{\prime }}=\;\frac{{\text{Fm}}^{\prime }-F^{\prime }}{{\text{Fm}}^{\prime }}$$

F′ is the initial fluorescence intensity and Fm′ is the maximum intensity under saturating light in culture adapted to 90 µmol photons m^−2^ s^−1^.

The Fv/Fm, RLCs and NPQ measurements were performed at *t* = 0 h and 4 ± 1 h in triplicate.

Three 5 mL sub-samples were dark-adapted for 1 h, allowing the full oxidation of PSII reaction centers and electron transport chain [2,3], before every measurement.

2. Fv/Fm were measured similarly than Fq′/Fm′ but on dark adapted sub-samples:
  (2) $$\frac{\text{Fv}}{\text{Fm}}=\;\frac{\text{Fm}-F0}{\text{Fm}}$$

F0 is the initial fluorescence intensity and Fm is the maximum intensity under saturating light in one hour dark adapted culture.

3. The RLCs were performed with seven incremental irradiance steps (10, 20, 50, 100, 300, 500 and 1000 µmol photons m^−2^ s^−1^) of 60 s. Physiological parameters were estimated by adjusting the model by Platt et al (1980) to the experimental data:
  (3) $$\text{rETR}\left(I\right)=\text{rETRmax}\;\times \;\left(1-e^{\left(-\alpha \;\times \frac{I}{\text{rETRmax}}\right)}\right)$$

rETRmax (AU) is the maximum relative electron transport rate and alpha (*α*) the initial slope of the RLC at limiting irradiance. The light saturation index Ek (µmol photons m^−2^ s^−1^) was then calculated following the equation:

(4) $$\text{Ek}=\;\frac{\text{rETRmax}}{\alpha }$$

4. NPQ induced by the RLC was calculated to assess the effect of the different microalgal extracts on the capacity of a targeted species (e.g. *D. salina*) to protect its reaction centers to an excess of light. NPQ induced by the rapid light curve was calculated according to the Stern Volner NPQ [15] and according to the following equation:
  (5) $$\text{NPQ}\;\text{induc}\;=\;\frac{\left(\text{Fm}-{\text{Fm}}^{\prime }\right)}{{\text{Fm}}^{\prime }}$$

Where Fm is the maximum fluorescent yield and Fm′ the maximum fluorescent yield in actinic light measured at the final RLC step.

## Results validating the method

**Fig. 3:**
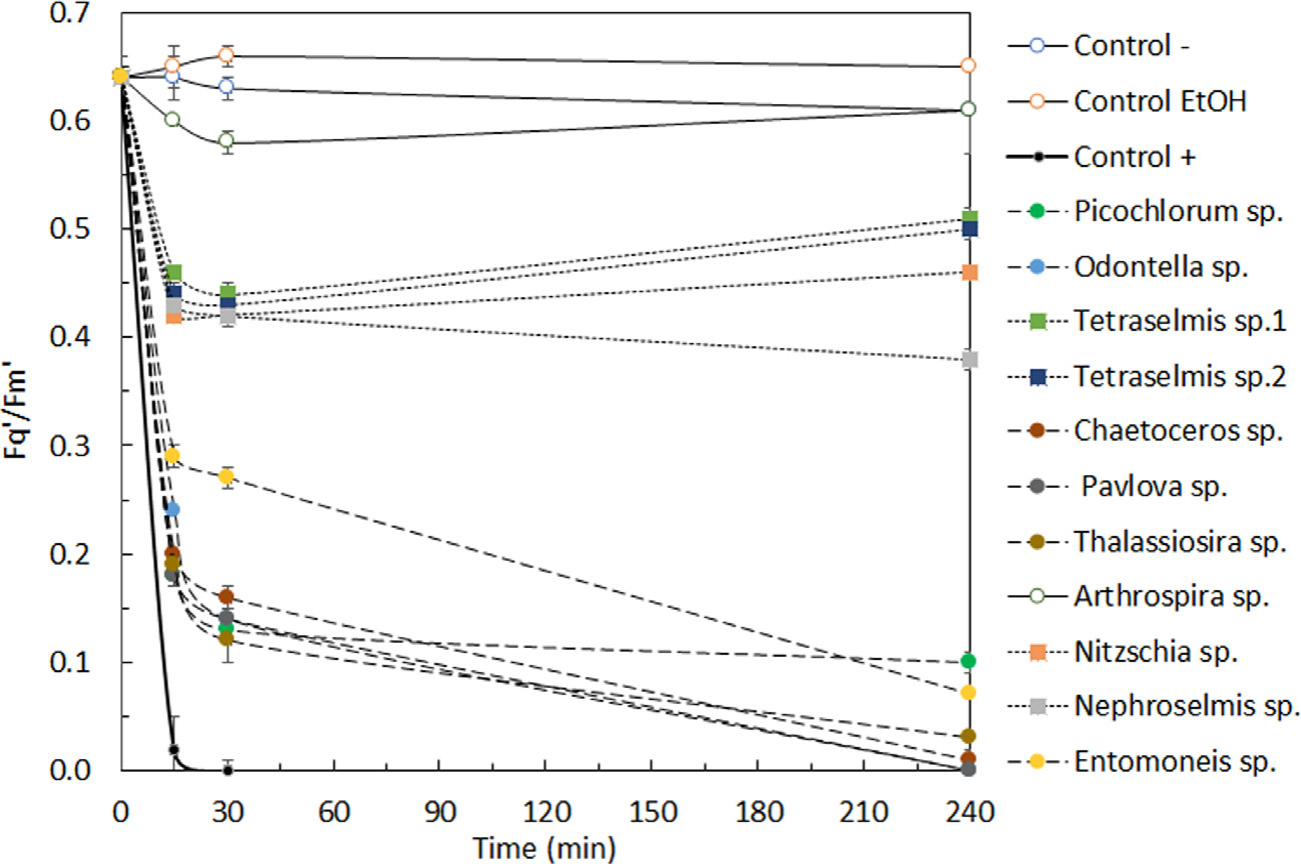
Effective quantum efficiency of the photosystem II (Fq′/Fm′, *n* = 3) during the experiment for *Dunaliella salina* exposed to different control conditions and different microalgal extracts.

**Fig. 4:**
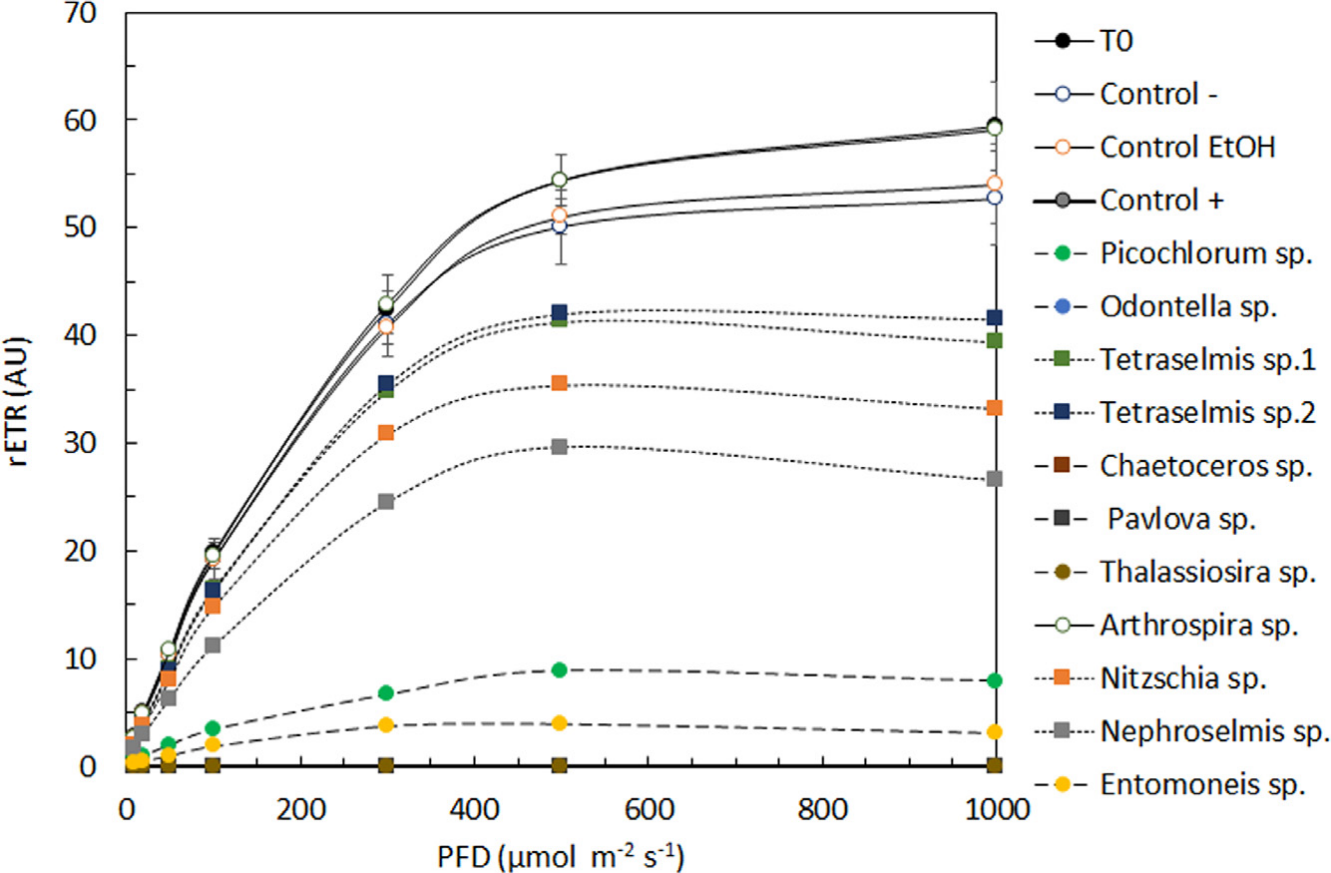
Rapid light curves (RLC, *n* = 3) expressed as the relative electron transport rate (rETR) as a function of the photon flux density (PFD in μmol photons m^−2^ s^−1^) for *Dunaliella salina* exposed to different control conditions and different microalgal extracts.

At stationary phase, the production in stirred closed PBR operating in continuous allows to produce *D. salina* at a stable photophysiological state over the period (10 days in this study) necessary to assess the impact of the different microalgal extracts. The initial Fq′/Fm′, Fv/Fm (*n* = 30) and RLC parameters (*n* = 15) were stable over time (The initial point in Fig. 3, and T0 in Fig. 4 and Table 1).

**Table 1:** Maximum quantum efficiency of the photosystem II (*Fv/Fm*) and rapid light curve (RLC) parameters after dark adaptation for *Dunaliella salina* exposed to different control conditions and different microalgal extracts. rETRm (AU) is the maximum relative electron transport rate. Alpha is the initial slope of the RLC at limiting irradiance. Ek (μmol photons m^−2^ s^−1^) is the light saturation coefficient. rETRm, alpha and Ek were estimated and non photochemical quenching induced (NPQ induc) by the RLCs were calculated according to the Stern Volner NPQ [8].

Condition	*Fv/Fm*	rETRm	Alpha	Ek	NPQ induc
T0	0.64 ± 0.03	61.70 ± 5.41	0.24 ± 0.03	255.21 ± 56.01	0.72 ± 0.10
Control -	0.63 ± 0.03	57.33 ± 2.51	0.25 ± 0.01	228.44 ± 10.84	0.68 ± 0.09
Control EtOH	0.64 ± 0.01	55.76 ± 3.61	0.24 ± 0.01	228.31 ± 21.55	0.65 ± 0.13
Control +	0.00	0.0	0.00	0.0	0.03 ± 0.03
*Picochlorum* sp.	0.13 ± 0.01	8.5	0.05	184.4	0.30
*Odontella* sp.	0.00	0.0	0.00	0.0	0.05
*Tetraselmis* sp.1	0.51 ± 0.01	41.7	0.23	181.0	0.48
*Tetraselmis* sp.2	0.50 ± 0.01	43.5	0.22	193.3	0.46
*Chaetoceros* sp.	0.00	0.0	0.00	0.0	0.10
*Pavlova* sp.	0.00	0.0	0.00	0.0	0.07
*Thalassiosira* sp.	0.00	0.0	0.00	0.0	0.12
*Arthrospira* sp.	0.61 ± 0.01	61.0	0.25	246.3	0.80
*Nitzschia* sp.	0.46 ± 0.01	35.4	0.21	168.0	0.30
*Nephroselmis* sp.	0.37 ± 0.01	28.8	0.16	177.1	0.34
*Entomoneis* sp.	0.065 ± 0.01	3.6	0.03	125.5	0.13

The use of PAM fluorometry allows to quickly assess the impact of the different microalgal extracts on the effective quantum efficiency of the PSII of *D. salina* as in 15 min, clear differences were observed between the different extracts tested to assess the method (Fig. 3).

The RLCs carried out after 4 h of exposure to the different microalgal extracts (Fig. 4) confirmed the results found using solely the Fq′/Fm′. In the present example with *D. salina* three different groups are clearly observed: (1) no impact (e.g. *Arthrospira* sp. and negative controls), (2) small inhibition (*Tetraselmis* sp. 1 and 2, *Nitzschia* sp. and *Nephroselmis* sp.) and (3) rapid and complete to almost complete inhibition of the PSII (*Picochlorum* sp., *Entomoneis* sp., *Thalassiosira* sp., *Chaetoceros* sp. *Pavlova* sp. and the positive control).

By adjusting the experimental data to fit the model of Platt et al. [16], different RLC parameters were calculated to assess the impact of the microalgal extracts on *D. salina* photophysiological parameters. In the present case, it allows to see clear differences on the rETRm and alpha, which can be considered as a proxy of the photosynthetic activity and as the capacity of the microalgae to carry out photosynthesis at limiting irradiance.

Similarly, the impact on NPQ was calculated to assess the capacity of *D. salina* to protect its reaction centers to an excess of light after exposition to the different microalgal extracts.

## Conclusion

The main advantages of this method are that:

1. Microalgal production at stationary phase in stirred closed PBR operating in continuous have stable photophysiological parameters, which is a necessary prerequisite to compare the impact of different algicidal compounds over time.
2. Pulse amplitude modulated fluorescence measurement is a non-destructive, non-invasive, fast and reliable method.
3. The combination of both methods, allow to quickly assess the algicidal effect of diverse microalgal (crude) extracts on the PSII efficiency of a tested microalgae and to avoid bias due to microalgae produced under unbalanced culture conditions (batch culture).
4. It may be used to identify and isolate algicidal molecules affecting algal PSII using a bio-guided isolation protocol.
5. The method could be generalized to all natural extracts to assess their algicidal effect.

## Declaration of Competing Interest

The authors declare that they have no known competing financial interests or personal relationships that could have appeared to influence the work reported in this paper.
